# Leveraging family dynamics to increase the effectiveness of incentives for physical activity: the FIT-FAM randomized controlled trial

**DOI:** 10.1186/s12966-020-01018-2

**Published:** 2020-09-10

**Authors:** Eric Andrew Finkelstein, Robyn Su May Lim, Dianne Stanton Ward, Kelly R. Evenson

**Affiliations:** 1 Programme in Health Services and Systems Research, Duke-NUS Medical School, 8 College Road, Singapore, 169857 Singapore; 2 Center for Health Promotion and Disease Prevention, University of North Carolina Chapel Hill, 1700 Martin Luther King Jr. Boulevard #7426, Chapel Hill, NC 27514 USA; 3 Department of Nutrition, Gillings School of Global Public Health, University of North Carolina Chapel Hill, 135 Dauer Drive, Chapel Hill, NC 27599 USA; 4 Department of Epidemiology, Gillings School of Global Public Health, University of North Carolina Chapel Hill, Chapel Hill, NC 27599-8050 USA

**Keywords:** Physical activity, Incentives, Group, Rewards, Randomized controlled trial, Activity tracker, Accelerometer, Parents, Children, Steps

## Abstract

**Background:**

Insufficient physical activity is a global public health concern. Research indicates incentives can increase physical activity levels of children but has not tested whether incentives targeted at children can be leveraged to increase physical activity levels of their parents. This study evaluates whether a novel incentive design linking children’s incentives to both their and their parent’s physical activity levels can increase parent’s physical activity.

**Methods:**

We conducted a two-arm, parallel, open-labelled randomized controlled trial in Singapore where parent-child dyads were randomly assigned to either (1) rewards to child contingent on child’s physical activity (child-based) or (2) rewards to child contingent on both child’s and parent’s physical activity (family-based). Parents had to be English-speaking, computer-literate, non-pregnant, full-time employees, aged 25–65 years, and with a participating child aged 7–11 years. Parent-child dyads were randomized within strata (self-reported low vs high weekly physical activity) into study arms in a 1:1 ratio. Participants were given activity trackers to assess daily steps. The outcome of interest was the between-arm difference in the change from baseline in parent’s mean steps/day measured by accelerometry at months 6 and 12 (primary endpoint).

**Results:**

Overall, 159 and 157 parent-child dyads were randomized to the child-based or family-based arms, respectively. Outcomes were evaluated on an intent-to-treat basis. At month 6, there was a 613 steps/day (95% CI: 54–1171) differential in favour of family-based parents. At month 12, our primary endpoint, the differential was reduced to 369 steps/day (95% CI: − 88–1114) and was no longer statistically significant.

**Conclusions:**

Our findings suggest that novel incentive designs that take advantage of group dynamics may be effective. However, in this design, the effectiveness of the family-based incentive to increase parent’s physical activity was not sustained through one year.

**Trial registration:**

NCT02516345 (ClinicalTrials.gov) registered on August 5, 2015.

## Background

There is overwhelming evidence that sustained physical activity reduces risks for non-communicable diseases, increases longevity, and reduces medical costs and productivity losses [1, 2]. Yet, the prevalence of insufficient physical activity has been steadily rising in high income countries, from 31·6% in 2001 to 36·8% in 2016 [3]. This has translated into global annual costs of roughly $54 billion and $14 billion in medical expenditures and productivity losses, respectively [4]. Thus, governments, insurers, and employers alike share a common interest in increasing physical activity.

Despite the obvious health benefits, there are many barriers to engaging in physical activity. One framework to consider how much to exercise is through the lens of classical economic theory [5]. This theory assumes that individuals are rational and weigh the costs and benefits of their decisions, including the decision of how much to exercise. If the benefits of an additional unit of exercise outweigh the costs, then the individual is expected to engage in the physical activity, otherwise s/he will not. This theory posits that one way to increase physical activity is through the use of economic incentives. Incentives, by increasing the benefits, are expected to induce greater levels of physical activity. Moreover, this finding holds even in the presence of several common biases, such as present-bias where individuals place too much weight on the immediate costs of exercise and too little weight (from their future selves’ perspective) on the potential health benefits that may not materialize until well into the future [6–8]. Consistent with these predictions, studies indicate that economic incentives can effectively increase physical activity in both children and adults [9–14].

Although studies have shown that incentives can be used to increase physical activity levels of the individual receiving the incentive, no studies to our knowledge have attempted to leverage incentives to increase physical activity of an affiliate, such as a close friend or family member, even if the affiliate is not a target of the incentive. Yet, economic theory further suggests this is possible if there is a cost, which need not be monetary, to the affiliate when the recipient does not obtain the reward and/or if the affiliate sees value in the recipient gaining the reward [15].

We tested this hypothesis using a two-arm, parallel, open-labelled randomized controlled trial (RCT) among child (recipient) and parent (affiliate) dyads. Focusing on child-parent dyads (term family-based) is appealing because children have been shown to increase physical activity levels even for relatively modest rewards [10] and because parents may be inclined to increase their physical activity levels both to not bear the “cost” of disappointing their child and because they value the increase in their child’s physical activity levels. We hypothesized that, for the same step targets and incentive level, the family-based incentive scheme would be more effective at increasing parent’s physical activity at months 6 and 12 (primary) than incentivizes that target children only (termed “child-based”).

## Methods

### Study design, recruitment, and participant characteristics

FIT-FAM (Financial Incentive Trial targeting FAMilies) was a 12-month (48-week), open-labelled RCT conducted in Singapore comparing two parallel arms (1:1 allocation ratio): (1) activity tracker plus child-based incentive, and (2) activity tracker plus family-based incentive. This manuscript conforms to CONSORT reporting guidelines (Additional file 1, CONSORT checklist).

Participants were recruited directly through advertisements and indirectly via “cold calls” to select companies. Ultimately, 5 private sector and 5 public sector companies agreed to participate and promote the study to their employees. All interested individuals recruited through the advertisements or from the company worksites were directed to the study website for additional information, registration, and eligibility screening, which was administered through an online questionnaire.

To be eligible, parents had to be English-speaking, computer-literate, non-pregnant, maintain full-time employment in Singapore, aged 25–65 years upon enrolment, and have a child aged 7–11 years who was willing to participate. We focused on parents who were full-time employees as this is a particularly inactive group in Singapore [16]. Both parent and child had to be able to climb ≥10 stair steps without stopping to minimize health concerns with participation. They also had to be willing to wear an activity tracker throughout the study and an accelerometer for 7 days each at baseline, month 6, and month 12. At baseline, all participants were required to provide ≥600 min/day of accelerometer wear time on at least 3 weekdays and 1 weekend day out of the 7 days before they were allowed to participate in the study.

Eligible parents wishing to participate signed an informed consent form, assented to their child’s participation, and paid a non-refundable enrolment fee of SGD25 (≈USD18.00). The nominal enrolment fee served as a deterrent to those who may join the study solely to receive the free activity tracker but who are not truly motivated to change their behavior. Those who answered ‘yes’ to any of the 7 Physical Activity Readiness Questionnaire questions, had a body mass index (BMI) of > 40 kg/m^2^, asthma, chronic obstructive pulmonary disease, diabetes mellitus, hypertension, lipid disorders, stroke, or personal or familial history of cardiovascular conditions were required to obtain written approval from a physician prior to enrolment.

### Randomization

**Fig. 1:**
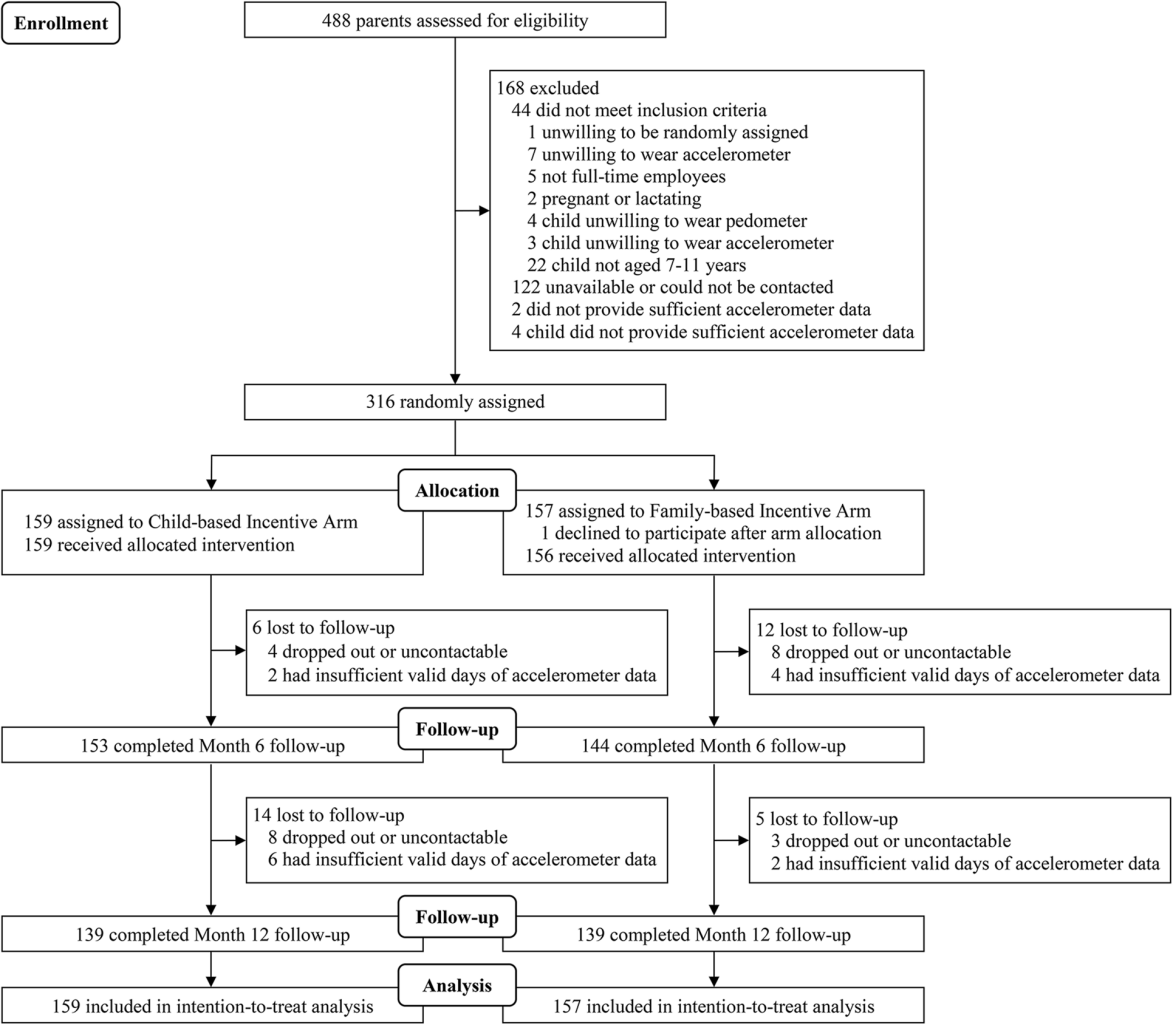
CONSORT diagram of FIT-FAM study parents

After completing enrolment, parent-child dyads were randomized with equal probability into one of two arms (Fig. 1, CONSORT flow diagram) within strata using a computer algorithm with strata defined by low or high amounts of weekly self-reported physical activity. Those who self-reported less than 60 min/week of moderate-to-vigorous physical activity (MVPA) were classified as low physical activity participants. A statistician generated the randomization list and did not disclose the allocation sequence to the study team members (research assistants/associates) involved in enrolling participants, revealing arm allocation, and delivering the interventions. For allocation concealment, sequentially numbered, opaque, and sealed randomization envelopes was used for the randomization assignment for all participants.

### Intervention

Upon enrolment, all participants were provided with booklets covering the benefits of and strategies for increasing physical activity, encouraged to achieve ≥10,000 steps/day (step target), and issued an activity tracker (Fitbit Zip® for children and a Fitbit Flex® for parents). The child’s activity tracker could be upgraded to a Fitbit Flex® for an additional SGD20 (≈USD14.40). Participants also had access to all features available on the Fitbit app and website.

### Child-based incentive arm

Children in the child-based study arm were awarded SGD5 (≈USD3.60) each week that they achieved the target through logging their steps on the activity tracker according to the following schedule: ≥10,000 steps/day on ≥4, ≥5, and ≥ 6 days each week in months 1–3, 4–6, and 7–12, respectively, *and* their participating parent logged ≥2000 steps/day on ≥4 days in the same week. The very low threshold for child-based parents, was meant to motivate child-based parents to wear the activity tracker. Children in the child-based arm could also earn a SGD5 (≈USD3.60) monthly bonus if they and their parents met their respective step targets in all 4 weeks in the month. This reinforcement strategy was used to encourage children to maintain streaks in efforts to promote a habit of sustained physical activity.

We chose a 10,000 steps/day target for simplicity, because this is an often-recommended target [17] and because it is the level advocated on the Fitbit app used by study participants [9]. Increasing the number of days required to reach the step goal throughout the study further encouraged participants to increase physical activity over time.

We chose the amount of SGD5 (≈USD3.60) for the weekly and monthly bonus incentives based on a prior study where children were rewarded a comparable amount for achieving monthly physical activity targets [10] and to accommodate the smallest voucher denomination carried by some providers. The maximum pay-out possible per child (in either arm) was valued at SGD300 (≈USD216) over 12 months (48 weeks).

### Family-based incentive arm

Family-based children were awarded SGD5 (≈USD3.60) each week that they *and* their participating parent achieved the same step target schedule presented to children in the child-based study arm through logging their steps on their activity trackers. Family-based children were also eligible to earn the monthly bonus if they and their participating parent met the goal in all weeks in the month.

For both arms, if either child or parent did not reach their weekly goal, the child earned no incentive for that week. Pay-outs were disbursed as child-friendly gift vouchers (e.g., Toys”R”Us).

### Outcomes and assessments

Step-tracking and awarding of incentives were based on the Fitbit activity trackers provided upon enrolment. However, to ensure higher quality data and to minimize missingness, the outcome of interest was the between-arm difference in the change in parents’ steps/day at months 6 and 12 (month 12 as primary endpoint) relative to baseline measured using waist-worn triaxial GT3X+ or wGT3X-BT ActiGraph accelerometers. Accelerometry was also used to measure the following secondary outcomes: steps/day (children); MVPA and MVPA bouts; sedentary duration; light, moderate, and vigorous physical activity; and total volume of physical activity (sum of light, moderate, and vigorous physical activity) presented in average minutes/day. Accelerometer data were expressed as average vector magnitude (VM) counts per minute (cpm). Tri-axial VM cut-points classified time in sedentary (0–199 cpm), light (200–2689 cpm), moderate (2690–6166 cpm), and vigorous (≥6167 cpm) minutes/day [18, 19]. MVPA was defined as VM ≥2690 cpm. MVPA bouts were defined as a total of 10 or more consecutive minutes above the MVPA VM cut-point with allowance for interruptions of 1 or 2 min below the cut-point [20]. The Choi et al. algorithm was used to identify adherent days [21]. A sample rate of 30 Hz and an epoch duration of 60 s were specified.

Participants were encouraged to wear the accelerometer on their waist for 7 days during waking hours at each assessment period, regardless of whether they wore their activity tracker during follow-up. Similar to the baseline assessment, data were considered adherent if ≥3 weekdays and 1 weekend day were provided for ≥600 min/day of wear time during each follow-up assessment period. All data were processed with R (version 3.5.1).

Secondary health outcomes for parents include BMI (Seca 217 Portable Stadiometer and Seca 869 Floor Scale), systolic blood pressure (Welch Allyn Spot Vital Signs Blood Pressure monitor), estimated cardiorespiratory fitness [maximum oxygen consumption (VO_2max_) approximated based on age, gender, BMI, resting heart rate (Welch Allyn Spot Vital Signs Blood Pressure monitor), and a non-exercise test (NET-F) for cardiorespiratory fitness; termed NET-F VO_2max_] [22, 23], and health-related quality of life (EQ-5D-5L) using the Thailand EQ-5D-5L value set as no Singapore value set was available [24]). All health outcome measures, excluding those collected through online questionnaires, were obtained at Duke-NUS Medical School or at the company worksites. Covariates (age, gender, and ethnicity) were captured at baseline and potential moderators were captured at baseline and both follow-up assessments using online questionnaires. For moderators, family dynamics were measured using the Family Adaptability and Cohesion Scale (FACES IV) [25], and parents’ social support for and enjoyment of physical activity were measured by the Physical Activity and Social Support scale (PASS) [26, 27] and the Physical Activity Enjoyment Scale (PACES), respectively [28]. To encourage attendance and questionnaire completion, parents were compensated SGD20 (≈USD14.40) per completed follow-up assessment.

### Sample size calculation

The study was powered to detect a medium effect size (r) of 0.3 between family-based and child-based parents assuming 20% attrition at month 12. We chose to power the study on a medium effect size so any differences observed would be both clinically meaningful and statistically significant. We were guided by effect sizes of this magnitude based on our prior studies [9, 10]. Using this effect size, an alpha of 0.05, and power of 0.8, we needed 158 parent-child dyads per arm.

### Statistical analysis

We hypothesized that the family-based incentive scheme would be more effective at increasing steps at months 6 and 12 (primary) [hypothesis (H) 1] and other measures of physical activity and health outcomes (H2) among parents without reducing children’s physical activity (H3). We also hypothesized that parents in the family-based incentive scheme would be more likely to achieve the weekly step targets (goal attainment) over the course of the study (H4).

We used a mixed effects linear difference-in-differences regression to test our primary hypothesis (H1) on parents with the key dependent variable being the number of daily steps recorded for person *i* on day *j* as measured by the accelerometer during each assessment. Independent variables include time and treatment (family-based participants) dummies and their interactions, controls for age, gender, and ethnicity. We also include fixed effects for worksites, random effects for individual participants, and adjusted the standard errors for clustering within individuals across days. Tests of our hypotheses were the sign and significance of the interaction terms, which allowed for testing whether step changes from baseline at months 6 and 12 (primary) were greater for family-based than child-based parents.

Analogous regressions were run for secondary hypotheses with the exception of H4. This hypothesis was tested with separate mixed effects logistic regression models for each incentive period where the dependent variable is an indicator variable for whether the ≥10,000 steps/day target was achieved and the key independent variable is a dummy for family-based participants. This variable allowed for testing whether those in the family-based arm were more likely to achieve the ≥10,000 steps/day targets in each incentive period. The logistic regressions included the same controls, and fixed and random effects as the linear model. Finally, we explored several potential moderators of effectiveness, including parent’s and child’s gender, family dynamics, and parent’s social support for and enjoyment of physical activity as described in Additional file 2. All analyses were from the intent-to-treat basis and conducted in Stata (version 14.2).

## Results

### Participant characteristics

Overall, 316 dyads were recruited from January 2016 to July 2017, of which 159 were randomly assigned to the child-based arm and 157 to the family-based arm (Fig. 1, CONSORT flow diagram). Parents were on average 42 years old (SD: 4.4) and 57.0% were male (Table 1). The majority were Chinese (75.3%), college graduates or postgraduates (85.4%), and, among those who declared, had monthly household incomes of ≥SGD10,000 (≈USD7,198) (31.1%). Children were on average 9 years old (SD: 1.4) and 54.8% were male.

**Table 1:** Participants’ baseline characteristics by study arm

Participant characteristics	All Participants (***n*** = 316)	Child-Based Incentive (***n*** = 159)	Family-Based Incentive (***n*** = 157)
Parents’ age, years, mean (SD)	42.0 (4.4)	42.0 (4.4)	42.1 (4.5)
Children’s age, years, mean (SD)	9.0 (1.4)	9.0 (1.4)	9.0 (1.4)
Parents’ sex, no. (%)			
Male	180 (57.0)	92 (57.9)	88 (56.1)
Children’s sex, no. (%)			
Male	173 (54.8)	94 (59.1)	79 (50.3)
Parents’ ethnicity, no. (%)			
Chinese	238 (75.3)	120 (75.5)	118 (75.2)
Malay	4 (1.3)	3 (1.9)	1 (0.6)
Indian	62 (19.6)	31 (19.5)	31 (19.8)
Other	12 (3.8)	5 (3.1)	7 (4.5)
Parents’ education, no. (%)			
High school or lower	3 (1.0)	0 (0.0)	3 (1.9)
Diploma or professional qualification	43 (13.7)	15 (9.4)	28 (17.8)
College graduate or higher	269 (85.4)	143 (89.9)	126 (80.3)
Monthly household income, no. (%)			
< SGD5,000	35 (11.1)	17 (10.7)	18 (11.5)
SGD5,000–SGD9,999	94 (29.8)	52 (32.7)	42 (26.8)
≥ SGD10,000	98 (31.1)	47 (29.6)	51 (32.5)
Don’t know	3 (1.0)	0 (0.0)	3 (1.9)
Prefer not to say	85 (27.0)	42 (26.4)	43 (27.4)

At baseline, parents in both arms exhibited similar numbers of steps/day with child-based and family-based parents logging 8425 (SD: 2807.1) and 8250 (SD: 2564.9) steps/day as measured via accelerometry (Table 2). All other measures of parents’ physical activity and health outcomes were also similar across arms (Table 2).

**Table 2:** Parents’ mean (SD) baseline measures by study arm

	All Participants (***n*** = 316)	Child-Based Incentive (***n*** = 159)	Family-Based Incentive (***n*** = 157)
**Average Baseline Levels of Accelerometry Measures**			
Steps/day	8338 (2693.0)	8425 (2807.1)	8250 (2564.9)
MVPA^a^ min/day	44.8 (24.5)	45.6 (24.4)	43.9 (24.5)
MVPA^a^ bout min/ day	16.9 (18.6)	17.7 (18.7)	16.2 (18.4)
Sedentary behavior min/day	526.4 (108.4)	523.0 (107.4)	529.8 (109.2)
Light physical activity min/day	302.6 (82.4)	303.6 (88.2)	301.7 (75.9)
Moderate physical activity min/day	42.0 (22.7)	42.8 (22.2)	41.3 (23.2)
Vigorous physical activity min/day	2.7 (5.1)	2.8 (5.6)	2.6 (4.7)
Total volume of physical activity^b^ min/day	347.3 (86.3)	349.2 (93.5)	345.6 (78.3)
**Average Baseline Levels of Health Measures**			
Body mass index (kg/m^2^)	24.4 (3.6)	24.2 (3.7)	24.5 (3.5)
Systolic blood pressure (mm Hg)	115.1 (17.0)	115.5 (17.7)	114.7 (16.2)
NET-F VO_2max_^c^	30.7 (3.6)	31.0 (3.6)	30.5 (3.5)
EQ-5D Index^d^	0.95 (0.09)	0.96 (0.08)	0.95 (0.10)

^a^ MVPA is moderate-to-vigorous physical activity ^b^ Total volume of physical activity is the sum of light, moderate, and vigorous physical activity minutes

^c^ NET-F VO_2max_ is a measure of cardiorespiratory fitness assessed without lab-based exercise

^d^ EQ-5D is a standardized means of measuring health-related quality of life

Six (3.8%) parents in the child-based and 12 (7.7%) in the family-based arm were lost to follow-up at month 6. Twenty (12.6%) parents in the child-based and 17 (10.8%) in the family-based arms were lost to follow-up at month 12 (Fig. 1).

### Steps logged and goal attainment as measured by the activity tracker

**Fig. 2:**
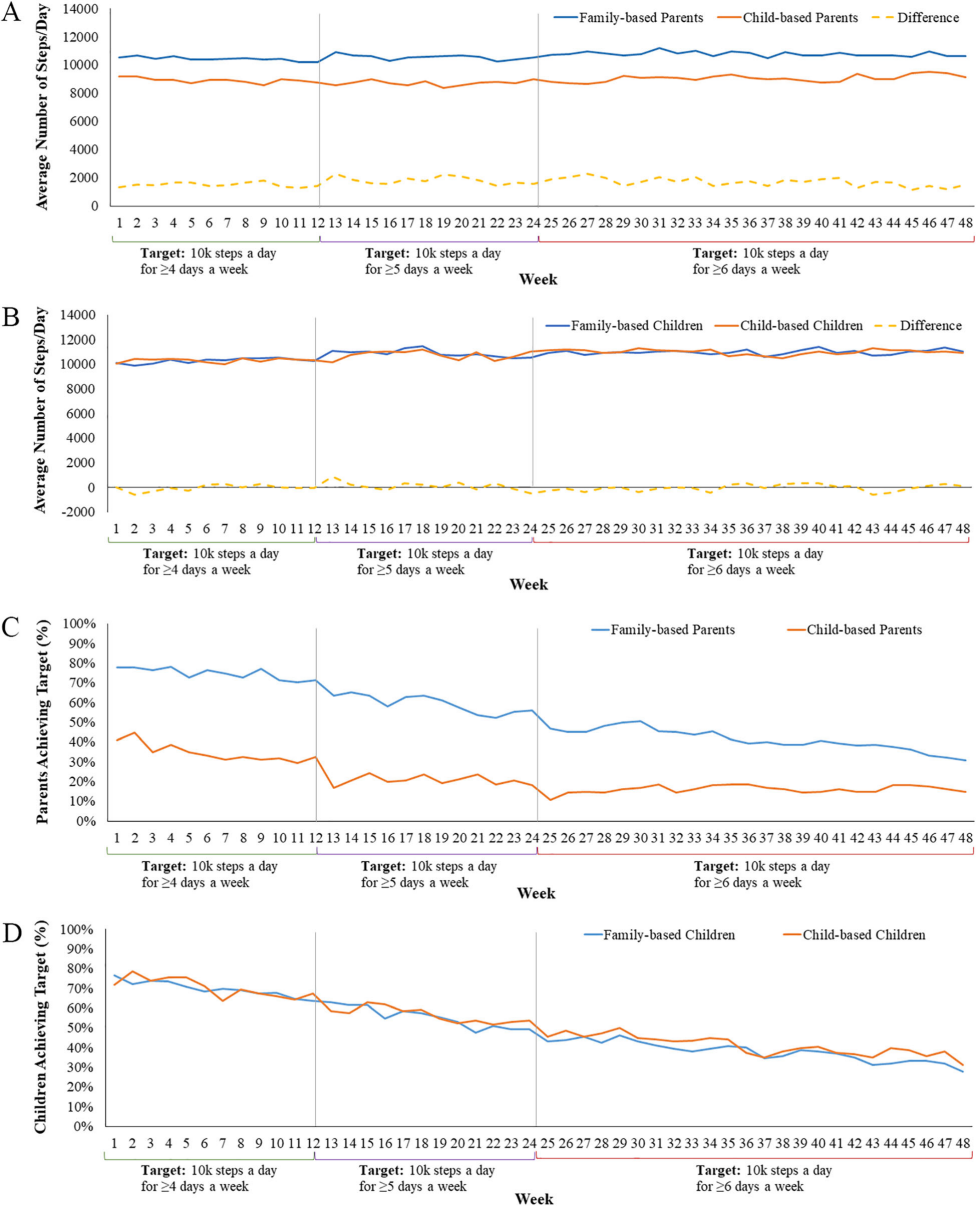
Average steps/day logged on activity trackers worn by (**a**) parents and (**b**) children and the percentage of (**c**) parents and (**d**) children meeting weekly step targets by arm over 48 weeks for days. when ≥500 steps (indicating more than minimal wear) were taken

Figure 2a and b show average steps/day logged on the activity tracker by week over 48 weeks for parents and children, respectively. Child-based parents and family-based parents logged on average 8955 steps/day, and 10,645 steps/day, respectively. Child-based and family-based children logged similar average steps/day of 10,772 and 10,793, respectively.

Fig. 2c and d show the percentage of parents and children meeting the step targets according to the ≥10,000 steps/day schedule by week over 48 weeks. Child-based parents met the target steps for 37, 23, and 21% of the possible weeks in months 1–3, 4–6, and 7–12, respectively. These same measures for meeting the target steps were higher (79, 67, and 55%, respectively) for parents in the family-based arm. Logistic regression results indicated statistically significant differences across all three incentive periods (Additional file 3) consistent with H4. Children in the child-based arm met the step target for 75, 65, and 55% of the possible weeks in months 1–3, 4–6, and 7–12, respectively. These same measures for meeting the target steps were similar to those of children in the family-based arm at 77, 67, and 57%, respectively. Logistic regression results revealed no statistically significant differences between the two arms (Additional file 4).

On average, children in the child-based and family-based arm earned SGD11.51 (≈USD8.29) and SGD11.30 (≈USD8.13) per month, respectively, over the 12-month incentive period.

### Parents’ outcomes

Regression results based on accelerometry at each assessment period are shown in Table 3. Wear time was not statistically different across arms (Additional file 5). At month 6, family-based parents increased their steps/day by 234 steps (95% CI: − 157–624) relative to baseline, whereas child-based parents decreased their steps/day by 379 steps (95% CI: -778–19). Consistent with H1, family-based parents had a positive and statistically significant step differential of 613 steps/day (95% CI: 54–1171) compared to child-based parents at month 6. Adjusting for wear time did not change the direction or significance of this result.

**Table 3:** Difference (95% CI) in parents’ accelerometer-derived outcomes at months 6 and 12^a^

	Difference from Baseline, by Study Arm		Between-arm Comparisons
	Child-Based Incentive (*n* = 159)	Family-Based Incentive (*n* = 157)	Family-Based Incentive vs Child-Based Incentive
**MONTH 6**			
Steps/day	−379 (−778, 19)	234 (− 157, 624)	613* (54, 1171)
MVPA^b^, min/day	− 0.2 (− 4.4, 3.9)	2.8 (− 0.7, 6.2)	3.0 (− 2.4, 8.4)
MVPA^b^ bouts, min/day	0.0 (− 3.2, 3.1)	3.5* (0.5, 6.5)	3.5 (− 0.8, 7.9)
Sedentary behavior, min/day	8.6 (−7.7, 25.0)	12.9 (−4.1, 29.8)	4.2 (− 19.3, 27.7)
Light physical activity, min/day	−12.9 (− 27.4, 1.7)	−15.8** (− 27.2, − 4.3)	−2.9 (− 21.4, 15.6)
Moderate physical activity, min/day	0.2 (− 3.5, 4.0)	2.4 (− 0.8, 5.5)	2.1 (− 2.8, 7.0)
Vigorous physical activity, min/day	− 0.5 (− 1.3, 0.4)	0.4 (− 0.7, 1.5)	0.9 (− 0.5, 2.2)
Total volume of physical activity^c^, min/day	− 13.1 (− 28.9, 2.7)	−13.0* (− 25.1, − 0.9)	− 0.1 (− 19.8, 20.0)
**MONTH 12**			
Steps/day	−208 (− 620, 203)	160 (− 219, 539)	369 (−191, 928)
MVPA^b^, min/ day	0.7 (−3.6, 5.1)	2.9 (− 0.5, 6.3)	2.2 (− 3.4, 7.7)
MVPA^b^ bouts, min/day	0.6 (− 2.6, 3.8)	2.4 (− 0.3, 5.2)	1.8 (− 2.4, 6.1)
Sedentary behavior, min/day	11.8 (−6.6, 30.3)	13.2 (− 6.7, 33.2)	1.4 (− 25.7, 28.5)
Light physical activity, min/ day	−14.5* (− 28.7, − 0.2)	− 16.5** (− 28.8, − 4.3)	−2.1 (− 20.9, 16.7)
Moderate physical activity, min/day	0.8 (−3.1, 4.7)	2.1 (−1.0, 5.2)	1.3 (− 3.7, 6.3)
Vigorous physical activity, min/day	0.01 (−1.0, 1.0)	0.8 (− 0.1, 1.6)	0.8 (− 0.5, 2.1)
Total volume of physical activity^c^, min/day	− 13.7 (− 28.4, 1.1)	− 13.6* (− 26.5, − 0.8)	0.0 (− 19.6, 19.6)

^a^ Unadjusted for wear time since wear time was comparable across arms ^b^ MVPA is moderate-to-vigorous physical activity ^c^ Total volume of physical activity is the sum of light, moderate, and vigorous physical activity minutes Note: * *p* < 0.05, ** *p* < 0.01, *** *p* < 0.001

At month 12, our primary endpoint, the family-based parents increased their steps/day by 160 steps (95% CI: − 219–539) relative to baseline whereas child-based parents decreased their steps/day by 208 steps (95% CI: − 620–203). However, the differential of 369 steps/day (95% CI: − 191–928), our primary outcome, was no longer statistically significant and inconsistent with H1. None of the secondary accelerometry-based measures (Table 3) nor any of the health outcomes (Table 4) were statistically different across arms at months 6 or 12, inconsistent with H2.

**Table 4:** Difference (95% CI) in parent’s health outcomes at months 6 and 12

	Difference from Baseline, by Study Arm		Between-arm Comparisons
	Child-Based Incentive (*n* = 159)	Family-Based Incentive (*n* = 157)	Family-Based Incentive vs Child-Based Incentive
**MONTH 6**			
Body mass index (kg/m^2^)	0.0 (− 0.1, 0.1)	0.0 (− 0.1, 0.1)	0.0 (− 0.1, 0.2)
Systolic blood pressure (mm Hg)	−1.1 (− 2.7, 0.4)	−1.7* (− 3.1, − 0.3)	− 0.6 (− 2.6, 1.5)
NET-F VO_2max_^a^	− 0.1 (− 0.2, 0.1)	−0.1* (− 0.2, 0.0)	0.0 (− 0.2, 0.1)
Change in EQ-5D Index^b^	0.016* (0.002, 0.031)	0.001 (− 0.015, 0.018)	−0.015 (− 0.037, 0.007)
**MONTH 12**			
Body mass index (kg/m^2^)	0.2* (0.0, 0.3)	0.2** (0.0, 0.3)	0.0 (−0.2, 0.2)
Systolic blood pressure (mm Hg)	−1.1 (− 2.9, 0.7)	−0.7 (− 2.3, 0.9)	0.4 (− 2.0, 2.8)
NET-F VO_2max_^a^	− 0.2*** (− 0.3, − 0.1)	− 0.2** (− 0.3, − 0.1)	0.0 (− 0.1, 0.2)
Change in EQ-5D Index^b^	−0.009 (− 0.025, 0.008)	−0.011 (− 0.026, 0.005)	−0.002 (− 0.024, 0.021)

^a^ NET-F VO_2max_ is a measure of cardiorespiratory fitness assessed without lab-based exercise

^b^ EQ-5D is a standardized means of measuring health-related quality of life

Note: * *p* < 0.05, ** *p* < 0.01, *** *p* < 0.001

### Children’s outcomes

Additional file 6 presents children’s accelerometer results. At month 6, relative to baseline, family-based children achieved a statistically significant increase of 464 steps/day (95% CI: 34–895), whereas children in the child-based study arm logged a decrease of 8 steps/day (95% CI: − 445–428), resulting in a statistically non-significant differential of 473 steps/day (95% CI: − 139–1085) between the two arms.

At month 12, relative to baseline, family-based children logged an increase of 315 steps/day (95% CI: − 131–761), while children in the child-based study arm logged an increase of 254 steps/day (CI: − 184–693), for a non-statistically significant differential of 61 steps/day (95% CI: − 565–686). Results for other children’s outcomes were also not statistically different across arms (Additional file 6). Hence, consistent with H3, the family-based incentive did not negatively affect the physical activity of children in the family-base arm.

### Potential moderators

Neither gender, social support for physical activity, family dynamics, nor enjoyment of physical activity moderated the effectiveness of the family-based incentives on steps (Additional file 7).

## Discussion

Prior studies have shown that incentives have been used successfully to influence health behaviors of the incentive target, ranging from physical activity, which is the focus of this effort, to smoking cessation, weight loss, medication adherence, and others. Classical economic theory posits that incentives *can be* effective to influence health behaviors, as long as the size of the incentive is large enough such that the expected benefits outweigh the costs of the behavior change. Behavioral economists and psychologists argue that it is not just the size of incentives that matters. They posit that, due to the presence of cognitive biases, many design features are likely to influence effectiveness, including type, frequency, and duration of incentive payments, and that taking advantage of these factors can increase effectiveness [29]. There is an additional literature arguing that group based incentives *may* work better than individual incentives under the assumption that members will work harder to obtain a goal so as not to let down the other group members [30]. However, this strategy tends to outperform individual incentives only if group members act pro-socially (i.e., they care about the other group members), their behavior is easily observable, and if they believe that their behavior will influence the behavior of others, suggesting it is likely to work best for small tightly knit groups. There is some evidence supporting this approach [13, 31].

This study takes the group based incentive strategy a step further. Because parents are expected to act pro-socially in support of their child’s physical activity levels, we hypothesized that extending a proven incentive strategy to both the child (target) and parent’s (affiliate) physical activity could be effective even if the parent only indirectly benefits and/or does not want to bear the ‘cost’ of disappointing their child. Such a strategy has not been tested previously.

Our findings provide only suggestive evidence consistent with this hypothesis. Our primary outcome of parents’ steps/day at 12 months was slightly greater for parents in the parent-based arm but not statistically different across arms, nor were any of the secondary accelerometry-based measures or health outcomes variables significantly different. However, at month 6, family-based parents had a positive step differential of 613 steps/day (95% CI: 54–1171) compared to child-based parents. Moreover, throughout the entire study, family-based parents were statistically and far more likely to reach the weekly step target than child-based parents. This suggests that that parents were changing their behavior as a result of the incentive design. There is no evidence that the design negatively affects children’s behavior.

The lack of more compelling results does not condemn such a strategy. As noted earlier, classical economic theory suggests that incentives can be effective to influence health behaviors, not that they will be in all cases. Incentives need to be large enough such that that the expected benefit is greater than the expected cost. The behavioural economics literature offers additional stipulations [15]. Therefore, it is not surprising that although many studies have shown that incentives work to change health behaviours, there is also a large body of literature showing cases where they do not [29].

In our case, several factors may explain the lack of statistically significant step increases at month 12, our primary endpoint. Although we cannot rule out that parents do not act pro-socially when it comes to their children’s health, it may be that design features led to a lack of effectiveness of our primary outcome. For example, a larger incentive may have led to greater changes in behavior. Alternatively, had we kept the step target unchanged between months 6 and months 12 (i.e., ≥10,000 steps/day on ≥5 days/week) and/or increased the reward value, the month 6 results might have been sustained. In fact, waning effectiveness of incentives over time for health behavior change is not unique to this study [29]. Changing behavior to improve health is difficult and maintaining those changes has proven to be even more difficult, suggesting that a larger reward may be necessary to sustain any changes in behavior that occur in the short term as a result of incentives. Finally, although we measured clinical health improvements, it is possible that even if our primary outcome were statistically significant, a specific duration and intensity of physical activity would be required to translate into clinically relevant health improvements. Without these improvements, it will be difficult to justify the value of any incentive-based program to funders. All of these factors should be considered in the design of future studies. Future research could also explore differential effects by socioeconomic status. Low socioeconomic status individuals may be more motivated by the prospect of a reward but may also be more constrained in their ability to adjust their behavior.

### Strengths and limitations

The primary strength of this study was testing our novel incentive design via a 12-month RCT. Other strengths include using accelerometers to measure the primary outcome, two assessment time points, and a low attrition rate of 12% at our primary endpoint.

The primary weakness of our approach is that whereas positive results provide evidence consistent with the theory, negative results are more difficult to interpret; does one reject the theory or the incentive design? Our design is one of endless possible reward strategies. Although we relied on incentive levels successfully employed in a prior study and incentive targets consistent with recommendations, it is certainly possible that an alternative design might have produced superior results.

Other limitations include the use of Fitbit models that do not detect wear time and the use of identical cut-points for children and adults when classifying activity levels into light, moderate, and vigorous physical activity. Although some studies use different cut-points for children and adults, the fact that the activity tracker and accelerometer generated the same conclusions for children (i.e., no differences across arms), we do not see this as a significant concern. Finally, our study is based on an educated sample recruited in Singapore, a fairly walkable city-state but one that is hot and humid, so whether or not these results would replicate in other populations or locations is unknown.

## Conclusions

There is growing interest in identifying low-cost incentive strategies that can be used to reduce risk factors for chronic disease. Our findings suggest that novel incentive designs that take advantage of group dynamics may be effective. However, in this particular design, the effectiveness of the family-based incentive to increase parent’s physical activity was not sustained at the one-year follow-up period.

## Supplementary information

**Additional file 1.** CONSORT checklist.

**Additional file 2.** Moderation analysis methods.

**Additional file 3.** Mixed effects logistic regressions - parents (odds ratios, 95% CI).

**Additional file 4.** Mixed effects logistic regressions - children (odds ratios, 95% CI).

**Additional file 5.** Parental and child average accelerometer wear time (min/day) by study arm.

**Additional file 6.** Difference (95% CI) in child accelerometer-derived outcomes at months 6 and 12 (unadjusted for wear time since wear time was comparable across arms).

**Additional file 7.** Moderation analysis results.

**Additional file 8.** Pearson correlation analysis between accelerometer-logged steps and activity tracker-logged steps.

## Data Availability

De-identified data will be made available upon reasonable request to the corresponding author. An investigator who proposes to use the data must have approval from an Institutional Review Board, Independent Ethics Committee, or Research Ethics Board, as applicable, and execute a data use/sharing agreement with Duke-NUS Medical School.

## Abbreviations

BMI: Body mass index
cpm: Counts per minute
EQ-5D-5L: Health-related quality of life
FACES IV: Family Adaptability and Cohesion Scale
MVPA: Moderate-to-vigorous physical activity
NET-F: Non-exercise test for fitness
PACES: Physical Activity Enjoyment Scale
PASS: Physical Activity and Social Support scale
RCT: Randomized controlled trial
VM: Vector magnitude
VO_2max_: Maximum oxygen consumption
