# Supplementary material for: Leveraging family dynamics to increase the effectiveness of incentives for physical activity: the FIT-FAM randomized controlled trial
Source: Int J Behav Nutr Phys Act. 2020 Sep 10;17:113. doi: 10.1186/s12966-020-01018-2 (PMC7488241; doi:10.1186/s12966-020-01018-2)
Supplement: Supplementary file 2 — Additional file 2. Moderation analysis methods. [file 12966_2020_1018_MOESM2_ESM.pdf]

Additional file 2. Moderation analysis methods.

$$\Delta\, Average\ \frac{Steps}{day}_{i,j} = \alpha + \beta_1 Family - based + \beta_2 Moderator_j + \beta_3 Family - based \times Moderator_j + \epsilon_{ij}$$

We conducted moderation analysis using the equation above. Separate regressions were run for change at months 6 and 12 relative to baseline. The following moderators were independently tested: gender (1 = male; 0 = female) of child and parent, parent's social support for physical activity as measured by PASS [1 = high social support (4-5); 0 = medium to low social support (0-3)].[1,2] Where PASS ranged from 0 – 5, with higher scores reflecting high support. Family dynamics is self-reported by parents and measured using FACES IV Family Satisfaction Scale. FACES IV [1 = high family satisfaction score (40-50); 0 = moderate to low family satisfaction score (10-39)] where FACES IV raw scores ranges from 10 – 50, with higher scores reflecting higher levels of family satisfaction. Parent's enjoyment of PA is measured by PACES [1 = high enjoyment of PA (40-56); 0 = moderate to low enjoyment of PA (0-40)]. PACES scores range from 0 – 56 with higher values reflecting greater levels of enjoyment.[3,4]

References

1. Eyler AA, Brownson RC, Rebecaa JD, et al. Physical activity social support and middle- and older-aged minority women: results from a US survey. *Soc Sci Med* 1999;49:781-89.
2. Ariely D. Predictably Irrational: HarperCollins 2008.
3. Kendzierski D, DeCarlo KJ. Physical activity enjoyment scale: Two validation studies. *J Sport Exerc Psychol* 1991;13(1):50-64.
4. Mullen SP, Olson EA, Phillips SM, et al. Measuring enjoyment of physical activity in older adults: invariance of the physical activity enjoyment scale (paces) across groups and time. *Int J Behav Nutr Phys Act* 2011;8(1):108.
